# *Paraburkholderia tropica* Primes a Multilayered Transcriptional Defense Response to the Nematode *Meloidogyne* spp. in Tomato

**DOI:** 10.3390/ijms252312584

**Published:** 2024-11-23

**Authors:** Carolina González-Cardona, Walter Ricardo López, Juan Jovel, Mauricio Soto-Suárez, Nelson Ceballos-Aguirre

**Affiliations:** 1 Facultad de Ciencias Agropecuarias, Universidad de Caldas, Calle 65 No. 26-10, Manizales 170003, Caldas, Colombia; carolina.gonzalez@ucaldas.edu.co (C.G.-C.); juan.jovel@ucalgary.ca (J.J.); msoto@agrosavia.co (M.S.-S.); 2 Departamento de Física y Química, Facultad de Ciencias Naturales, Universidad Nacional de Colombia Sede Manizales, km 9 vía Aeropuerto la Nubia, Manizales 170003, Caldas, Colombia; wrlopez@unal.edu.co; 3 Faculty of Veterinary Medicine, University of Calgary, 3280 Hospital Dr NW, Calgary, AB T2N 4Z6, Canada; 4 Corporación Colombiana de Investigación Agropecuaria-AGROSAVIA, km 14 vía Mosquera-Bogotá, Mosquera 250047, Cundinamarca, Colombia

**Keywords:** root-knot nematode, RNA-seq, plant defense mechanisms, hydrolase activity

## Abstract

Meloidogyne causes a devastating disease known as root-knot that affects tomatoes and other cash crops worldwide. Conversely, *Paraburkholderia tropica* has proven beneficial in mitigating the effects of various pathogens in plants. We aimed to unravel the molecular events that underlie the beneficial effects of the bacterium and the detrimental impacts of the nematode when inoculated separately or together in tomato plants. The transcriptional responses induced by *P. tropica* (TB group (tomato-bacteria group)), *Meloidogyne* spp. (TN group (tomato-nematode group)) or by the two agents (TBN group (tomato-bacteria-nematode group)) in tomato were assessed by RNA-seq. We implemented a transcript discovery pipeline which allowed the identification of 2283 putative novel transcripts. Differential expression analysis revealed that upregulated transcripts were much more numerous than downregulated ones. At the gene ontology level, the most activated term was ‘hydrolase activity acting on ester bonds’ in all groups. In addition, when both microbes were inoculated together, ‘hydrolase activity acting on O-glycosyl compounds’ was activated. This finding suggests defense responses related to lipid and carbohydrate metabolism, membrane remodeling and signal transduction. Notably, defense genes, transcription factors and protein kinases stood out. Differentially expressed transcripts suggest the activation of a multifaceted plant defense response against the nematode occurred, which was exacerbated by pre-inoculation of *P. tropica*.

## 1. Introduction

Nematodes in the Meloidogyne genus cause a devastating disease typically known as root-knot. This name derives from the characteristic large galls these nematodes induce in the root system of infected plants [1]. Such galls affect the absorption of nutrients and water, hinder developmental processes and ultimately reduce both the yield and quality of crops [2,3,4]. Collectively, root-knot nematodes (RKNs) cause losses in a variety of cash crops worldwide that surpass USD 100 billion [3,5,6,7,8]. Although a multitude of management practices have been attempted to counter RKN infestations, none of them appears to be effective alone, and integrated approaches are therefore favored [9]. Even so, RKNs continue to be a recurrent phytosanitary threat, especially in tropical and subtropical areas of the world, in many crops, including tomatoes [10]. In the current global agricultural landscape, ideal novel management practices for RKNs should be environmentally friendly. In this context, biological agents such as bacteria that enhance plant growth and stress tolerance rank highly. These bacteria promote plant growth and yield through phosphate solubilization, nitrogen fixation and activation of plant defense mechanisms [11,12,13]. The application of bacteria as biological control agents of plant-parasitic nematodes has proven effective in diminishing nematode populations [14]. This efficacy is due not only to the aforementioned capabilities but also to parasitism and the production of bacterial toxins and antibiotics [15,16].

*Paraburkholderia tropica*, formerly *Burkholderia tropica*, is a rod-shaped, Gram-negative, motile, nitrogen-fixing, aerobic bacterium [17,18,19,20,21]. It was originally isolated from sugarcane plants in several tropical countries, including Brazil, Mexico and South Africa [22]. The growth-promoting ability of *P. tropica* has been well characterized and is attributed to its diazotrophic nature, which allows the conversion of environmental nitrogen into more assimilable forms such as ammonia [23,24]. Furthermore, it also acts as an iron-chelating agent (solubilizing ferric iron) through the production and secretion of siderophores [25,26] as well as in the production of exo-heteropolysaccharides [27,28]. In evaluations as a plant growth promoter, Paraburkholderia has proven to be promising in a variety of crops, including sugarcane, barley, tomato, sorghum and several tree species [21,25,26,29,30,31].

A number of mechanisms whereby Paraburkholderia species act as biological controllers of plant pathogens have been described. For instance, in several broccoli cultivars, it was shown that root inoculation with various Parabukholderia species induces metabolomic changes in the shoots and those changes correlate with resistance to the pathogen *Xanthomonas campestris*, which is indicative of the induction of systemic resistance. Moreover, a specific interaction between broccoli cultivars and Paraburkholderia species has been reported [32]. Mycophagy was demonstrated to occur with *Paraburkholderia busanensis* against *Colletotrichum scovillei*, a fungus that causes anthracnose in pepper cultivars. It was proposed that the molecular mechanism likely involved chitin and N-acetylglucosamine utilization genes found in the *P. busanensis* genome [33]. In maize, several strains of Paraburkholderia reduced the detrimental effects of *Fusarium oxysporum* and *F. verticilliodes*, and metabolomic analysis showed a correlation between antifungal activity and the components of the ornbactin and pyochelin siderophores [34]. Culture filtrates of *Burkholderia cepacia* assayed as a biological control of *Meloidogyne incognita* were shown to have an inhibitory effect on the egg hatching and motility of second-stage juveniles [35]. Similarly, a study conducted in RKN-infested soils in South Korea showed that Burkholderia (JB-2 strain) cell-free filtrates reduced the density of *M. incognita* and egg mass formation in tomato [36]. Therefore, Paraburkholderia shows potential as a biological agent for nematode management in tomato cultivation.

Reflecting its established ability to upregulate systemic defense genes in distant tissues, foliar applications of Paraburkholderia have also proven to be effective against diverse plant pathogens [37,38,39]. Preliminary transcriptomic studies in *Solanum torvum* infested with RKNs demonstrated that the activation of a number of genes involved in defense responses occurred, including lignin biosynthesis and cell wall modifications [40]. Analogous studies in rice identified a series of transcription factors associated with defense responses and hormone signaling pathways [41,42]. Combined metabolomic and RNAseq experiments in roots of *Phaseolus vulgaris* colonized by *Burkholderia phymatum* suggested that the regulation of host genes involved in nodulation was involved [43]. Dual transcriptomic analysis on sugarcane infected with *Paraburkholderia Q208* demonstrated that the activation of plant genes involved in metabolism occurred [44]. In rice, *Burkholderia vietnamiensis* and *Paraburkholderia kururiensis* inoculated to roots and leaves were shown to induce a jasmonic acid systemic response [45].

These results suggest a complex molecular interplay takes place between plants, pathogens and synergistic microorganisms such as *P. tropica.* In Colombian tomato fields, nematodes, mainly of the genus Meloidogyne, are important pathogens. The tropical weather favors the establishment of beneficial bacteria such as *P. tropica.* A pilot experiment inoculating this bacterium to the roots or to the foliage of tomato seedlings resulted in improved plant growth in the latter group. In order to assess defense responses induced by *Meloidogyne* spp. and *P. tropica*, we conducted the inoculation of both microorganisms separate or together in tomato and analyzed early molecular responses by RNAseq. To the best of our knowledge, this is the first study exploring the molecular interactions in the tomato–Meloidogyne–*P. tropica* complex.

## 2. Results

**Figure 1:**
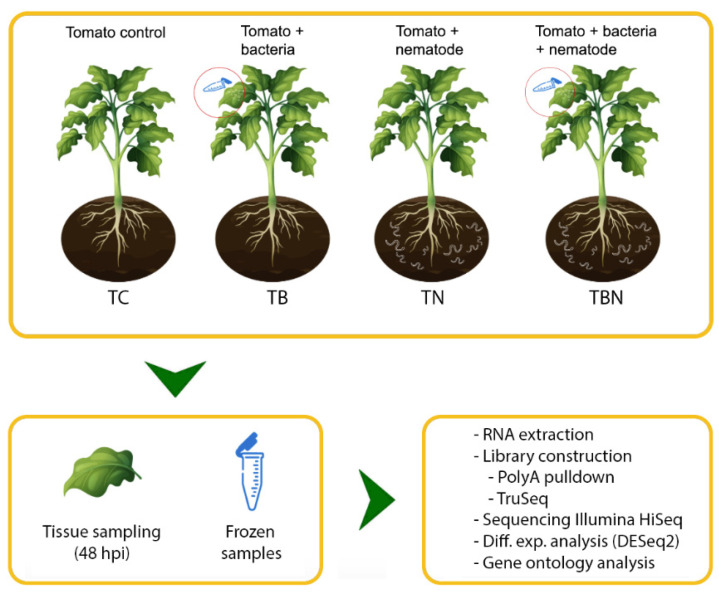
Description of experiment. Tomato plants (20 DAS) were either mock-inoculated (TC), inoculated with *Paraburkholderia tropica* (TB) on fully developed upper leaves, with *Meloidogyne incognita* (TN) to the radical area or with a mix of both (TBN). In the latter case, *P. tropica* was inoculated as above and 24 h later, the nematode was applied as before. In all cases, systemic leaves were collected 48 h after nematode inoculation (hpi) in the TBN group. Leaves were snap-frozen in liquid nitrogen upon collection and subsequently lyophilized. Lyophilized material was used for total RNA extraction and construction of RNAseq libraries.

Given that several species of Paraburkholderia have proven to be promising as biological control agents of diverse plant pathogens, including bacteria [32,46], fungi [33,34] and nematodes [35,36], we assessed the ability of *P. tropica* to induce systemic defense responses against the nematode *Meloidogyne* spp. (Figure 1).

To maintain conciseness, we will use the following abbreviations going forward: TC (tomato control plants), TB (tomato plants inoculated with *P. tropica*), TN (tomato plants inoculated with the nematode *Meloidogyne* spp.) and TBN (tomato plants inoculated with both *P. tropica* and *Meloidogyne* spp.) (Figure 1). In order to have a better chance of surveying transcripts that were expressed to only low levels, we sequenced each library to a depth ranging from 9–25 M reads (Supplementary Figure S1A). The average quality of libraries was remarkably high, usually with an average Q score above 35 (Supplementary Figure S1B). Nevertheless, reads were trimmed using a Q score threshold of 30, which resulted in the removal of only a marginal number of reads. Pairwise correlation of transcripts abundance in multi-library experiments is conventionally considered a proxy of library quality. Pearson correlation coefficients (*r*) greater than 0.8 are considered good, while coefficients greater than 0.9 are considered excellent. One library had a correlation coefficient with 0.83 < *r* < 0.88, while the rest were well above 0.9 (Supplementary Figure S2). Thus, the high quality and the depth of our libraries favor a thorough scrutiny of the tomato transcriptome when plants were exposed to *P. tropica*, *Meloidogyne* spp. or both.

### 2.1. Discovery of Putative Novel Transcripts

**Figure 2:**
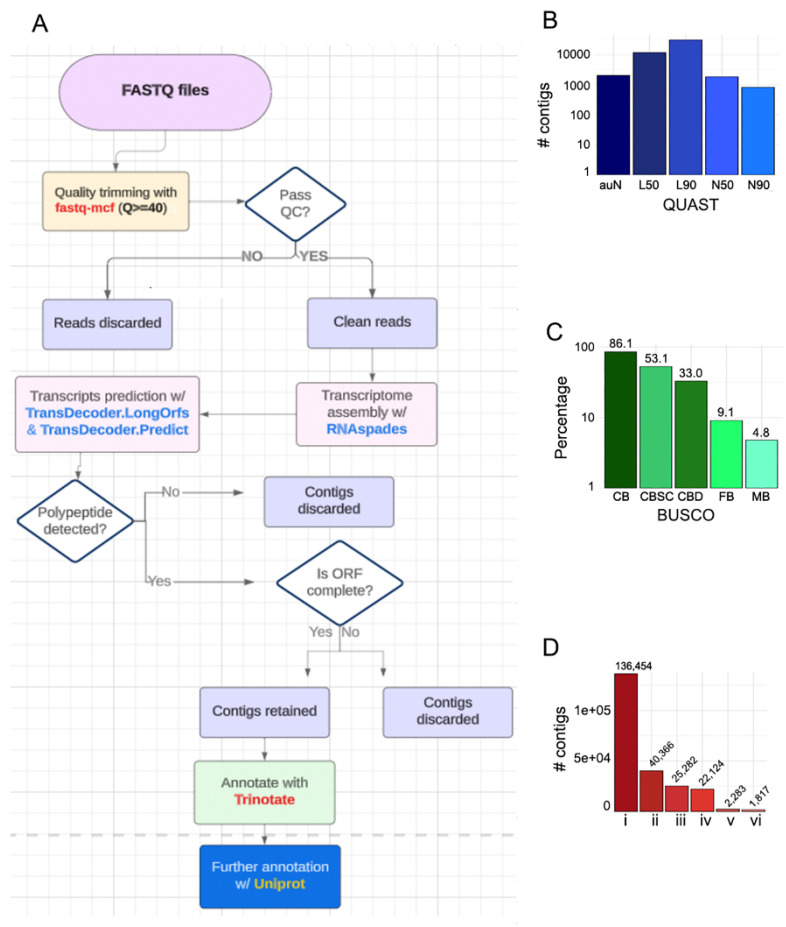
Assemble of putative novel transcripts. (**A**) Computational workflow. (**B**) Assembly statistics derived with QUAST (Quality Assessment Tool for Genome Assemblies). N50 and N90: Minimal length of scaffolds, whose sum equals 50% and 90% of the length of the genome, respectively. L50 and L90: This refers to the minimal number of contigs that are required to equal 50% and 90% of the genome length, respectively. auN: This reflects contiguity in the assembly. It is defined as the area under an Nx curve. It can be computed by multiplying the length of each contig (Li) by the proportion of the assembled genome it accounts for (Li/∑Li), then summing these values for all i contigs. (**C**) Assembly statistics derived with BUSCO (Benchmarking Universal Single Copy Orthologs). CB: Complete BUSCO orthologs, CBSC: Complete and single copy BUSCO orthologs, CBD: Complete and duplicated BUSCO orthologs, FB: Fragmented BUSCO orthologs, MB: Missing BUSCO orthologs. (**D**) Statistics of assembly. (i) Contigs assembled. (ii) Number of contigs that resulted in predicted partial or complete transcripts. (iii) Number of complete transcripts (with start and end codons). (iv) Number of transcripts that could be annotated. (v) Predicted transcripts that failed to align to the reference genome and are therefore considered novel transcripts. (vi) Number of unique proteins contained in the putative novel transcripts.

A significant part of the transcriptome is expressed facultatively in response to stimuli [47]. Although transcriptional alterations induced by *Meloidogyne* spp. infestations have been explored by RNAseq, much less is known about them in the case of *P. tropica* or the concurrent inoculation of both microbes in tomato. We reasoned that a set of transcripts specifically induced under such conditions might be lacking in the tomato reference transcriptome. We therefore put in place a transcript discovery pipeline to search for novel transcripts (Figure 2A).

We conducted a combined assembly whereby all libraries were concatenated and assembled with rnaSPAdes [48]. Assembly quality was assessed using QUAST (Quality ASsessment Tool for genome assembly) [49] and BUSCO (Benchmarking Universal Single-copy Orthologs) [50] and the results are presented in Figure 2B,C. Of special interest are the QUAST N50 and BUSCO CB (complete BUSCO) metrics, which have values of 2941 and 86.1%, respectively. This indicates that 50% of the total assembly was included in contigs with a minimal length of 2941 bp and 86.1% of single-copy and duplicated orthologs were included in our assembly. A total of 136,454 contigs longer or equal to 300 bp were obtained (Figure 2D). Such contigs were subjected to a two-step prediction of transcripts using the TransDecoder software. TransDecoder selects only contigs that contain open reading frames (ORF) of at least 100 amino acids and harbor typical protein domains. This yielded 40,366 putative transcripts. From those, partial transcripts comprising only 5′, 3′ or central moieties were discarded, leaving for annotation 25,282 putative transcripts that initiated with a start codon (ATG), were at least 100 amino acids in length and ended with a termination codon (Figure 2D). Such putative transcripts were functionally annotated with Trinotate [51]. A total of 22,124 putative transcripts were annotated. Annotated transcripts were aligned against the tomato ITAG4.1 transcriptome used as reference in this study and 5520 of them failed to align (Supplementary Table S1). To increase the stringency of our discovery pipeline, these transcripts were aligned to the tomato genome (ITAG4.0). Surprisingly, only 2283 (~42%) putative novel transcripts could be mapped to the tomato genome, while 3237 (~58%) failed to align. The mapped novel transcripts corresponded to 1817 different proteins and are referred to as novel transcripts (Figure 2D). The 2283 mapped novel transcripts with annotated sequences were concatenated with the ITAG4.1 transcriptome and used as the reference for transcript quantification and subsequent differential expression and gene ontology analyses.

### 2.2. Differentially Expressed Transcripts

Hereafter, the transcripts annotated by the international tomato genome sequencing project (transcriptome version ITAG4.1) and putative novel transcripts assembled as described above are discussed together. The identifiers (ids) of ITAG4.1 transcripts are prefixed by ‘Solyc’ while the ids of our putative novel transcripts are prefixed by ‘SL4.0’, which refers to the tomato genome version used for mapping the assembled putative transcripts. In Table 1, we present a quantitative summary of differentially expressed transcripts, as well as a qualitative description of the gene ontology terms found to be over- or under-represented during gene enrichment analysis.

When compared to control plants, the majority of transcripts with significant changes in expression were upregulated in the three comparisons (Supplementary Table S1). Their number was ascending from TB, to TN to TBN (Supplementary Tables S2–S4) with the highest number being in the double inoculation group.

**Figure 3:**
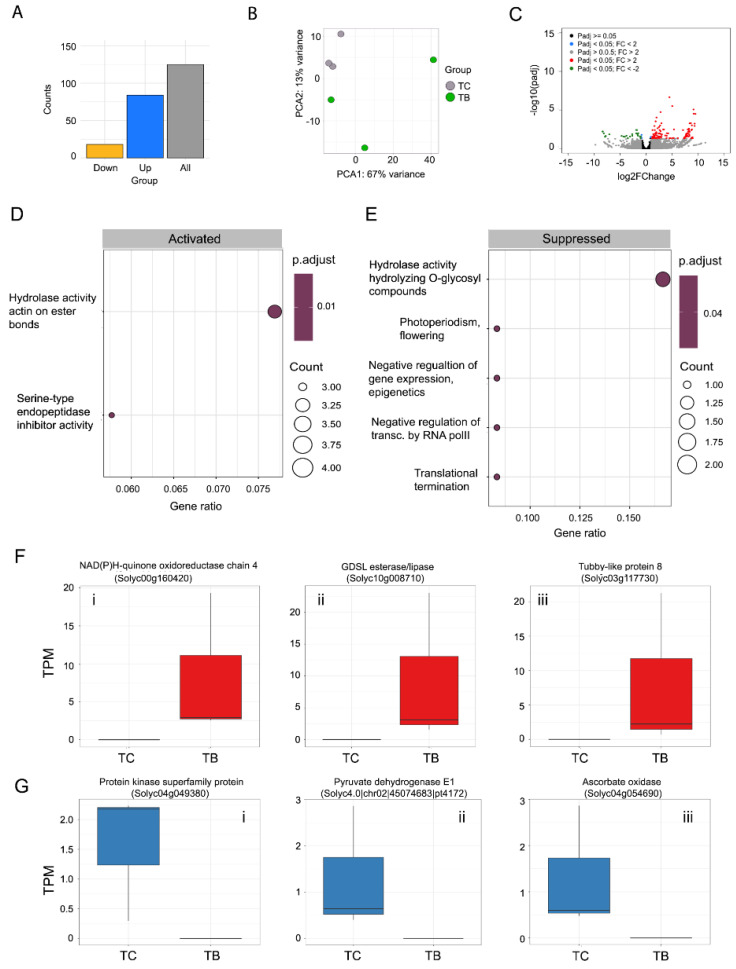
Differential expression analyses results for comparison TC vs. TB. (**A**) Number of differentially expressed transcripts (grey bar), number of upregulated transcripts (blue bar) and number of downregulated transcripts (yellow bar). (**B**) Principal component analysis (PCA) on Euclidean distances of count data subjected to regularized logarithmic transformation. (**C**) Volcano plot depicting differentially expressed genes. Red dots represent genes with a corrected *p*-value (padj) < 0.05 and a log2fold change ≥1. Green dots represent genes with a corrected *p*-value (padj) < 0.05 and a log2fold change ≤−1. All other dots are non-significant, as described in the legend. (**D**) Gene ontology terms that were found activated at a statistically significant level. (**E**) Gene ontology terms that were found suppressed at a statistically significant level. (**F**) Boxplots representing the normalized (TPM) abundance of the three most upregulated transcripts in the TC vs. TB comparison. (**G**) Boxplots representing the normalized (TPM) abundance of the three most downregulated transcripts in the TC vs. TB comparison.

**Figure 4:**
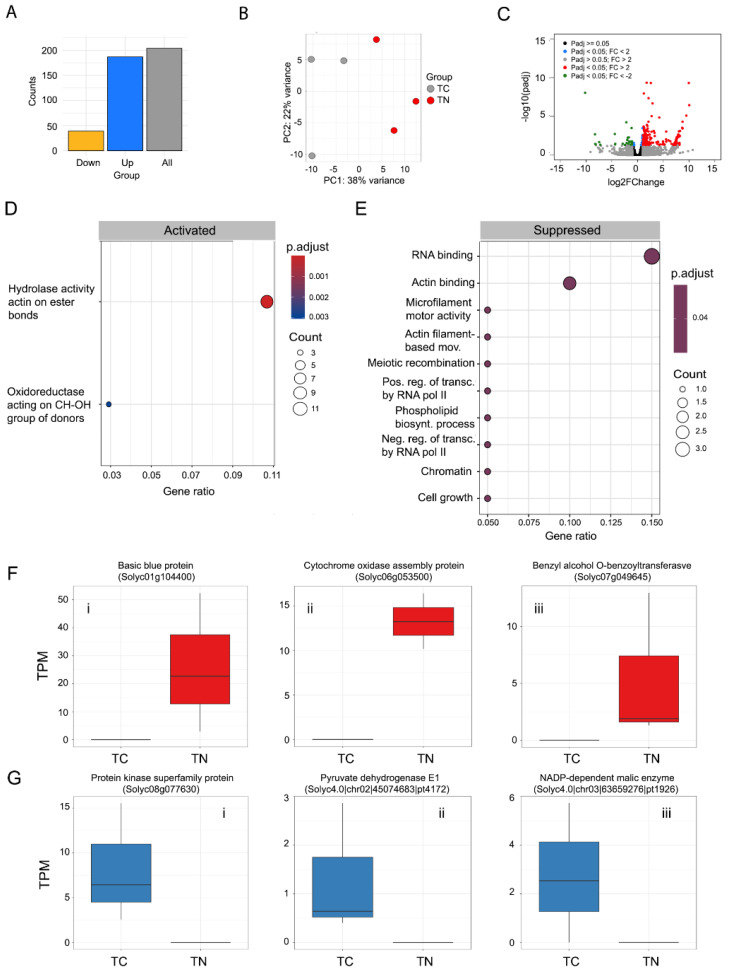
Differential expression analyses results for comparison TC vs. TN. (**A**) Number of differentially expressed transcripts (grey bar), number of upregulated transcripts (blue bar) and number of downregulated transcripts (yellow bar). (**B**) Principal component analysis (PCA) on Euclidean distances of count data subjected to regularized logarithmic transformation. (**C**) Volcano plot depicting differentially expressed genes. Red dots represent genes with a corrected *p*-value (padj) < 0.05 and a log2fold change ≥1. Green dots represent genes with a corrected *p*-value (padj) < 0.05 and a log2fold change ≤−1. All other dots are non-significant, as described in the legend. (**D**) Gene ontology terms that were found activated at a statistically significant level. (**E**) Gene ontology terms that were found suppressed at a statistically significant level. (**F**) Boxplots representing the normalized (TPM) abundance of the three most upregulated transcripts in the TC vs. TN comparison. (**G**) Boxplots representing the normalized (TPM) abundance of the three most downregulated transcripts in the TC vs. TN comparison.

**Figure 5:**
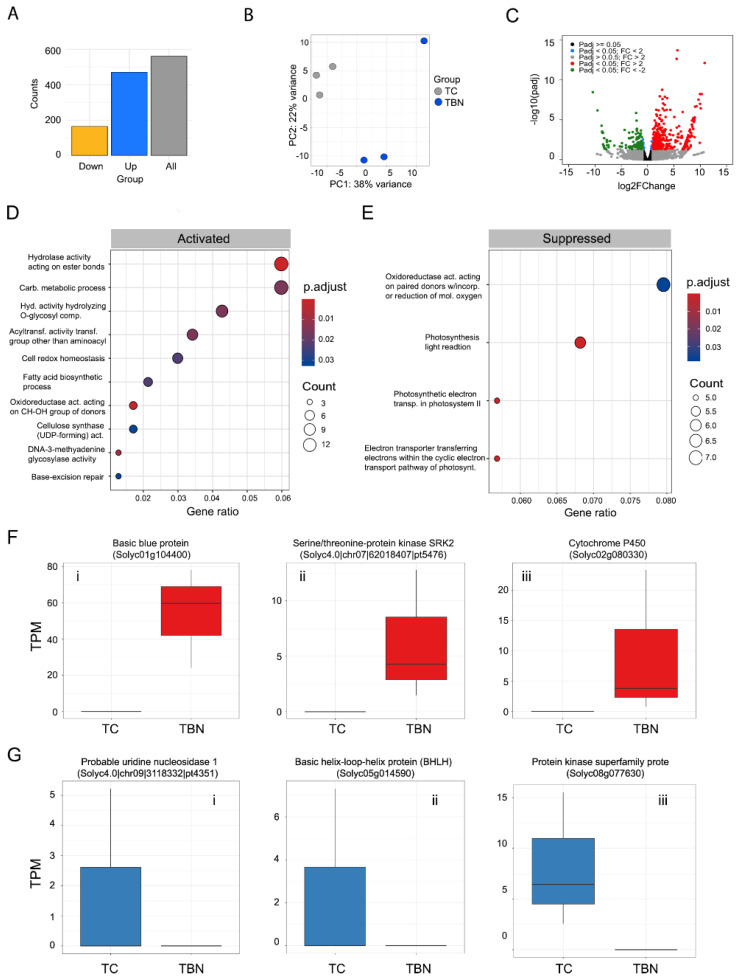
Differential expression analyses results for comparison TC vs. TBN. (**A**) Number of differentially expressed transcripts (grey bar), number of upregulated transcripts (blue bar) and number of downregulated transcripts (yellow bar). (**B**) Principal component analysis (PCA) on Euclidean distances of count data subjected to regularized logarithmic transformation. (**C**) Volcano plot depicting differentially expressed genes. Red dots represent genes with a corrected *p*-value (padj) < 0.05 and a log2fold change ≥1. Green dots represent genes with a corrected *p*-value (padj) < 0.05 and a log2fold change ≤−1. All other dots are non-significant, as described in the legend. (**D**) Gene ontology terms that were found activated at a statistically significant level. (**E**) Gene ontology terms that were found suppressed at a statistically significant level. (**F**) Boxplots representing the normalized (TPM) abundance of the three most upregulated transcripts in the TC vs. TBN comparison. (**G**) Boxplots representing the normalized (TPM) abundance of the three most downregulated transcripts in the TC vs. TBN comparison.

The number of transcripts up- and downregulated was, respectively, TB: 100 and 24 (Figure 3A; Supplementary Table S2); TN: 173 and 30 (Figure 4A; Supplementary Table S3); TBN: 414 and 146 (Figure 5A; Supplementary Table S4). Only the most up- and downregulated transcripts known to play a central role in plant defense against microbes will be discussed. For transcripts found differentially expressed, gene ontology analyses were conducted with clusterProfiler with a customized script that incorporates the gene ontology annotations obtained, for the novel transcripts, with the trinotate pipeline (Supplementary Tables S5–S8).

### 2.3. Transcriptional Plant Responses to P. tropica Alone

Principal component analysis (PCA) showed a clear separation between the control and *P. tropica*-inoculated plants, with higher variability observed in the later group (Figure 3B).

This finding indicated that application of *P. tropica* alone does alter the tomato transcriptome. After differential expression analysis, the number and the average log2fold change (log2FC) of upregulated transcripts were larger than in downregulated transcripts (Figure 3B,C; Supplementary Table S2).

In the context of gene ontology analysis, a term is activated or suppressed when the enrichment analysis of upregulated or downregulated transcripts, respectively, is statistically significant. The ‘hydrolase activity acting on ester bonds’ (GO:0016788) was the main process activated (Figure 3D), while ‘hydrolase activity hydrolyzing O-glycosyl compounds’ (GO:0004553) was the most suppressed term (Figure 3E). Other processes related to the negative regulation of gene expression (GO:0045814), negative regulation of transcription (GO:0000122) and translation termination (GO:0006415) were also found to be mildly suppressed, which suggests an increase in the synthesis of nucleic acids and proteins occurred.

At the individual transcript level, the three most upregulated transcripts upon *P. tropica* inoculation were *NAD(P)H-quinone oxidoreductase chain 4* (Solyc00g160420; log2FC: 9.46), *GDSL esterase/lipase* (Solyc10g008710; log2FC: 9.31) and *Tubby-like protein 8* (Solyc03g117730; log2FC: 9.30) (Figure 3F). The three most downregulated transcripts were *Protein kinase superfamily protein* (Solyc04g049380; log2FC: −8.37), *Pyruvate dehydrogenase E1 component subunit alpha, mitochondrial* (Solyc4.0|chr02|45074683|pt4172; log2FC: −8.15) and *Ascorbate oxidase* (Solyc04g054690; log2FC: 8.00) (Figure 3G).

### 2.4. Transcriptional Plant Responses to Meloidogyne spp. Alone

The transcriptional response induced by *Meloidogyne* spp. in tomato was quantitatively and qualitatively different from the one induced by *P. tropica.* The number of deregulated transcripts found in the presence of *Meloidogyne* spp. was almost twice the number deregulated by *P. tropica* (Figure 4A–C).

Curiously, as was observed in the TB group at the gene ontology level, ‘hydrolase activity acting on ester bonds’ (GO:0016788) was also the main process activated (Figure 4D). RNA binding (GO:0003723) and cytoskeleton proteins’ activity were the most significantly suppressed terms (Figure 4E). Moreover, processes related to nucleic acid and phospholipid synthesis as well as to cell growth appeared to be slightly suppressed (Figure 4E).

At the individual transcript level, the three most upregulated transcripts following *Meloidogyne* spp’s inoculation were *Basic blue protein* (Solyc01g104400; log2FC: 10.04), *Cytochrome oxidase assembly protein* (Solyc06g053500; log2FC: 9.94) and *Benzyl alcohol O-benzoyltransferase* (Solyc07g049645; log2FC: 9.46) (Figure 4F). The three most downregulated transcripts were *Protein kinase superfamily protein* (Solyc08g077630; log2FC: −10.19), *Pyruvate dehydrogenase E1 component subunit alpha, mitochondrial* (Solyc4.0|chr02|45074683|pt4172; log2FC: −8.27) and *NADP-dependent malic enzyme, chloroplastic* (Solyc4.0|chr03|63659276|pt1926; log2FC: 8.22) (Figure 4G).

### 2.5. Transcriptional Plant Responses Induced by P. tropica and Meloidogyne spp.

Interestingly, when *Meloidogyne* spp. inoculation was preceded by *P. tropica* inoculation, the number of deregulated transcripts was substantially higher than that found when each microbe was inoculated separately (Figure 5A). PCA plotting also showed a clear separation of transcriptomes between the control and double-inoculated plants (Figure 5B) with the majority of differentially expressed transcripts being upregulated (Figure 5C).

As found in the individual microbe inoculations, ‘hydrolase activity acting on ester bonds’ (GO:0016788) was also the main process activated, but additional enriched processes were also detected. Notably, ‘carbohydrate metabolic process’ (GO:0005975) and ‘hydrolase activity hydrolysing O-glycosyl compounds’ (GO:0004553), which was suppressed by *P. tropica* alone, appeared to be activated in TBN-inoculated plants. In addition, ‘acyltransferase activity, transferring groups other than amino-acyl groups’ (GO:0016747), ‘cell redox homeostasis’ (GO:0045454) and ‘fatty acid biosynthetic process’ (GO:0006633), among other biosynthetic processes, were also found to be activated (Figure 5D). The most suppressed term was ‘oxidoreductase activity acting on paired donors with incorporation or reduction of molecular oxygen’ (GO:0016709) as well as processes related to photosynthesis and electron transport (Figure 5E).

The most upregulated transcript in plants inoculated with both microbes was *Basic blue protein* (Solyc01g104400; log2FC: 10.84), followed by *Serine/threonine-protein kinase SRK2I* (Solyc4.0|chr07|62018407|pt5476; log2FC: 10.25) and *Cytochrome P450* (Solyc02g080330; log2FC: 10.09) (Figure 5F). The three most downregulated transcripts were a *Probable uridine nucleosidase 1* (Solyc4.0|chr09|3118332|pt4351; log2FC: −21.74), *Basic helix-loop-helix* (bHLH) DNA-binding superfamily protein (Solyc05g014590; log2FC: −21.71) and *Protein kinase superfamily protein* (Solyc08g077630; log2FC: 10.40) (Figure 5G).

### 2.6. Overlapping Differentially Expressed Transcripts Among Treatments

**Figure 6:**
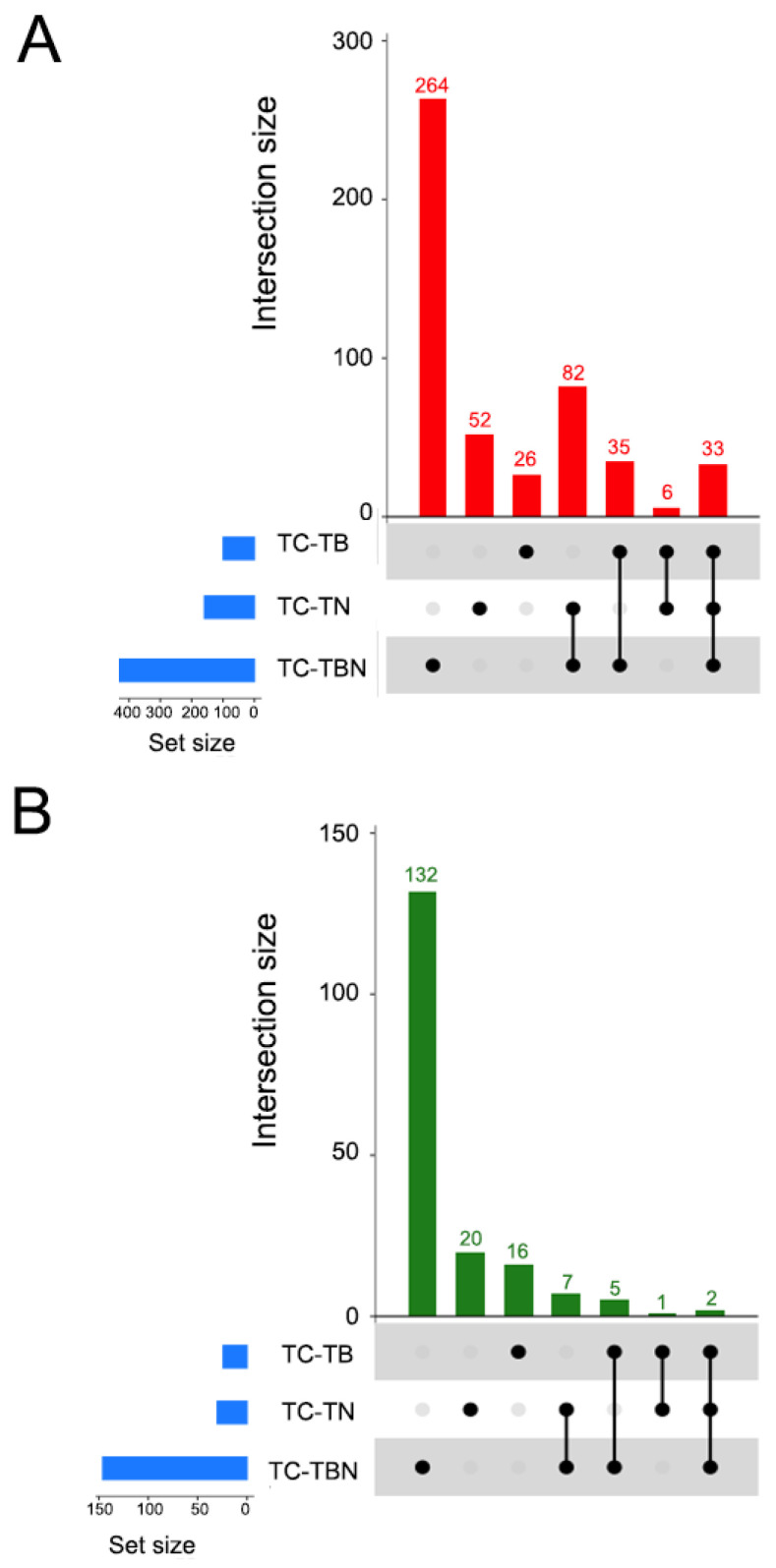
Intersection analysis of differentially expressed (DE) genes. (**A**) UpSet plot depicting the number of genes upregulated exclusively in each of the comparisons conducted, or in two or three comparisons. (**B**) UpSet plot depicting the number of genes downregulated exclusively in each of the comparisons conducted, or in two or three comparisons.

To facilitate the interpretation of differentially expressed transcripts, we conducted an intersection analysis, whereby transcript sets found differentially expressed in one or more comparisons are identified. The results are shown in UpSet plots (Figure 6A,B). For significantly upregulated transcripts, 333, 60 and 33 were unique to the TBN, TN and TB groups, respectively. There was an overlap between TN and TBN of 68 transcripts and of 35 transcripts between the TB and TBN groups. Strikingly, only seven transcripts were upregulated in the TN and TB treatments. Twenty-four transcripts were shared by the three groups. The pattern observed in the downregulated genes was similar to the one of the upregulated genes, but numbers were lower in each case. Remarkably, no transcript was commonly downregulated in the TB and TN treatments. Supplementary Tables S9–S24 descriibe transcripts that were found differentially expressed in more than one comparison, separately for upregulated and downregulated transcripts.

### 2.7. Protein Functional Categories Differentially Expressed

**Figure 7:**
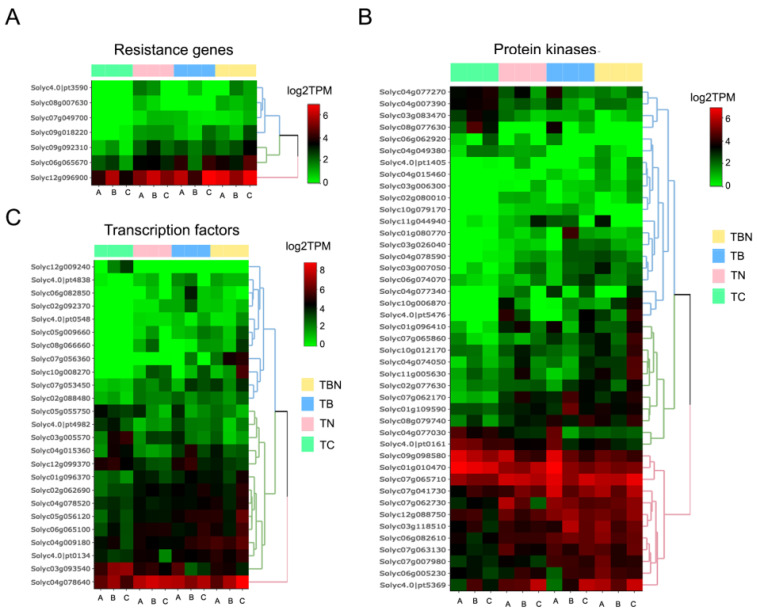
Normalized abundance of DE transcripts in key functional protein groups. (**A**) Relative abundance of DE transcripts involved in resistance. (**B**) Relative abundance of DE transcripts encoding protein kinases. (**C**) Relative abundance of DE transcripts encoding transcription factors. A, B, C labels at the bottom of each heatmap indicate individual replicates (plants) used for each treatment.

There are some protein functional categories among the differentially expressed transcripts that deserve special attention because they have the potential to be involved in response to nematode infection, the *P. tropica* growth-promoting process or both. These include, although are not limited to, resistance genes, transcription factors and protein kinases (Figure 7). For instance, when *Meloidogyne* spp. was inoculated alone, three resistance transcripts were found to be upregulated (*Disease resistance protein*, Solyc07g049700; *Tobacco mosaic virus resistance-2*, Solyc09g018220; and *Pleiotropic drug resistance protein 4*, Solyc06g065670). The last two transcripts were also found to be upregulated in the double inoculation (*P. tropica* and *Meloidogyne* spp.) with a higher fold change. Moreover, four additional transcripts belonging to resistance genes were also found to be upregulated in the double-inoculation group (*Disease resistance protein I2*, Solyc08g007630; *Putative late blight resistance protein homolog R1B-12*, Solyc4.0|chr05|4004717|pt3590; *Late blight resistance protein*, Solyc09g092310; and *Disease resistance protein (TIR-NBS-LRR class) family*, Solyc12g096900). None of those transcripts were significantly deregulated in plants inoculated with the bacteria *P. tropica* alone, although heatmaps depicting normalized abundance of transcripts also showed upregulation of resistance genes transcripts (Figure 7A and Figure S3).

Another group of transcripts found to be altered in their expression are transcription factors (TFs). Out of twenty-five differentially expressed transcripts encoding isoforms of TFs, eighteen were found to be upregulated and seven downregulated (Supplementary Figure S4). Three transcripts were found exclusively upregulated in the TB group (TFs GRAS, myelin, as well as an isoform of GATA). Four TFs were differentially expressed exclusively in the group TN: RAP2.1 and R2R3MYB were upregulated while two isoforms of GATA were downregulated. All other differentially expressed TFs were in the group TBN, either exclusively or shared with the TB or TN. Namely, the bHLH superfamily of TF were found to be dramatically upregulated in groups TN and TBN (log2FC > 8) and TF PERIANTH was found to be highly upregulated in groups TB and TBN (log2FC > 8). TF MYC was found moderately upregulated in all comparisons. WRKY, atf1 and LBM2 and two ethylene-responsive TFs were all downregulated in group TBN (Figure 7B and Figure S4).

The largest functional group differentially expressed in all treatments were transcripts encoding protein kinases (Figure 7C). Out of these, 34 were found upregulated and 11 downregulated. For groups TB, TN and TBN, the number of these transcripts encoding protein kinases were seven, five and thirty-nine, respectively. The most upregulated kinase was Serine/threonine-protein kinase (group TBN). In both the TN and TBN groups, Leucine-rich receptor-like protein kinase (log2FC > 7) and Phosphatidylinositol 4-phosphate 5-kinase (log2FC > 3) were largely upregulated. Another kinase domain protein (A0A200QFW1_9MAGN) was also found upregulated in all comparisons (log2FC > 3) (Supplementary Figure S5). Interestingly, several auxin responsive genes were found to be differentially expressed (see Supplementary Tables S2–S4 and Figure S5).

### 2.8. Differentially Expressed Novel Transcripts

Seventy-five novel transcripts were found to be differentially expressed (Supplementary Figure S6). In agreement with the general trend observed, most novel transcripts were upregulated. Five differentially expressed novel transcripts were kinases, four were upregulated and one was slightly downregulated. Four differentially expressed novel transcripts were transcription factors. One splicing factor was dramatically upregulated in group TN (log2FC > 7) and another was slightly upregulated in group TBN.

To gain additional insights into the expression level and magnitude of the deregulation of differentially expressed novel transcripts, we created MA plots for all differentially expressed transcripts to depict the relative expression levels (base mean) and the magnitude of deregulation (log2FC). For all comparisons, the novel transcripts’ base mean and log2FC (blue dots) were intermingled with ITAG annotated transcripts (red dots; Supplementary Figure S7A–C). Thus, differentially expressed novel transcripts showed a similar level of expression and change in expression level as reference ITAG transcripts. Moreover, since splicing is a widespread hallmark of eukaryotic transcription, we mapped the sequence of novel transcripts along the tomato genome and visualized such alignments. We visually inspected a few examples, and, in all cases, spliced alignments could be observed for differentially expressed novel transcripts in the three groups (TB, TN and TBN; Supplementary Figure S7D–F). In summary, several lines of evidence suggest that the novel transcripts reported here, specifically those found to be differentially expressed, appear to constitute integral parts of the tomato transcriptome.

### 2.9. Distribution of Differentially Expressed Transcripts Along Chromosomes

**Figure 8:**
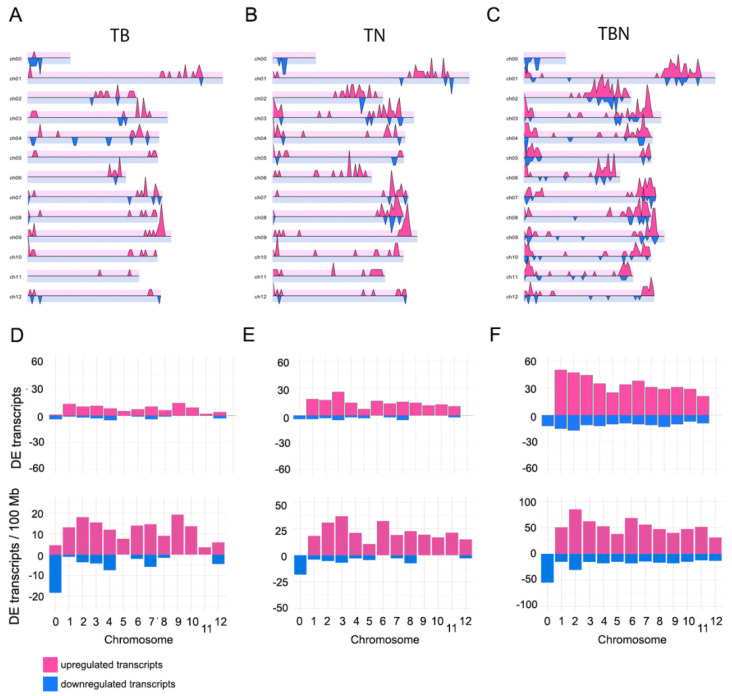
Distribution of genes found differentially expressed (DE) along the tomato chromosomes. Location of DE genes along tomato chromosomes for comparison control vs. bacteria (**A**), control vs. nematode (**B**) or control vs. bacteria + nematode (**C**). (**D**), (**E**) and (**F**) depict the number of DE genes in comparisons (**A**), (**B**) and (**C**), respectively. The upper panels correspond to counts of DE genes, while the lower panels represent normalized abundance of DE transcripts per 100 Mb. However, none of the tomato chromosomes are that long, so we used that scale to account for the relatively small number of DE transcripts per Mb. In all panels, pink and blue bars correspond to upregulated and downregulated genes, respectively.

At first glance, in a linear representation, the distribution of deregulated transcripts along the tomato genome appears to cluster at the terminal regions of the chromosomes, and more conspicuously on the positive strand and at the distal end of each chromosome. It was similar for the three groups (Figure 8A–C). Chromosomes 1, 2 and 3 accumulated the largest number of differentially expressed transcripts (Figure 8D–F, upper panels). This pattern was partially preserved when transcript density was normalized per chromosome length, although density in shorter chromosomes increased (Figure 8D–F, lower panels). To investigate if the noticeable accumulation of deregulated transcripts at the terminal region of the chromosomes was the result of nematode- and/or bacteria-specific clusters of defense genes, or only reflected the general density of genes along the tomato genome, we created plots for all the tomato genes along the chromosomes. The results clearly show that the spatial distribution of differentially expressed transcripts along the chromosomes in our experiment faithfully reflects the density of genes in the tomato genome (Supplementary Figure S8). To better characterize this observation, we conducted Pearson correlation analysis between the general distribution of genes on the tomato genome and the distribution of differentially expressed transcripts in each group. In all cases, Pearson correlation coefficients were very high and significant (Supplementary Figure S9).

## 3. Discussion

We sequentially inoculated *Paraburkholderia tropica* and *Meloidogyne* spp in tomato plants. This study hypothesized that *P. tropica’s* benefits on plant growth and stress tolerance might derive from the priming of plant resistance mechanisms against Meloidogyne, which would be detectable through RNA sequencing.

We initially implemented a transcript discovery pipeline in search of transcripts not included in the reference transcriptome (ITAG4.1) but induced under our experimental conditions [51,52]. We report here more than 2000 putative novel transcripts that encode a variety of functions. Given the rigor of our pipeline, we believe that at least a significant fraction of these transcripts are bona fide messenger RNAs, potentially enhancing our understanding of tomato molecular biology. *P. tropica* elicited a weaker transcriptional response than *Meloidogyne* spp., which is in line with the pathogenic nature of the latter. Sequential co-inoculation with *P. tropica* followed by the nematode triggered an amplified transcriptional response. One possible explanation is that the bacterium primed a defense response in the tomato plants.

The transcriptional responses included the activation of resistance (R) genes, which were not significantly upregulated in the TB group (bacterium-only inoculation) but were in the TN (nematode-only) and even more strongly in the TBN (co-inoculated) group. Although, to our knowledge, none of the resistance genes found upregulated here have been implicated in specific resistance against nematodes [1], it remains possible that they integrate a broad-spectrum resistance circuit. Paraburkholderia has been reported to prime R genes, including Mi-1, thus enabling a rapid response to invading RKNs [53,54], but that was not observed in our results. Dominant resistance (R) genes are cell surface or intracellular receptors [55] that mediate effector-triggered immunity (ETI) [56], which often culminates in hypersensitive cell death [57,58]. The experimental duration was insufficient to determine if a hypersensitive response was induced against the nematode. R genes are adapted to detect pathogen effectors, typically constituting variants of nucleotide-binding leucine-rich repeat receptors (NLRs) [55,59]. Little is known about R genes mediating systemic responses against RKNs. Although R genes have been discovered, most remain to be cloned and characterized [1]. A notable exception is the tomato *Mi1.2* R gene [60]. Similarly, little is known about RKN effectors [1].

Another prominent group of transcripts found differentially expressed were those encoding protein kinases, which are reported to play prominent roles in plant defense against pathogens [61]. Forty-four kinase transcripts were differentially expressed, with most of them showing upregulation. RKNs have been reported to activate protein kinases [42,62,63,64,65], and the Burkholderia lipopolysaccharide (LPS) stimulation of seedlings of *Arabidopsis thaliana* and *Nicotiana tabacum* also resulted in the activation of plant kinases [66,67], as did the inoculation of *Burkholderia* Q208 in sugarcane plants [44]. Kinases are closely related to pattern recognition receptors (PRRs) that are involved in recognition of pathogen-associated molecular patterns (PAMPs) that trigger immune responses [62,68] and promote synthesis of structural components like callose, lignin and secondary metabolites such as phenolic compounds [69]. In tomato plants inoculated with both organisms (TBN group), transcripts encoding Serine/threonine protein kinase SRK21 and Receptor-like protein kinase THESEUS were dramatically upregulated. In Arabidopsis, the THESEUS 1/FERONIA family has been reported to include cell wall sensors [70] and be involved in stress-related responses and modulation of mechanical properties of the cell wall, turgor loss point and ABA synthesis and function [71].

The transcriptional activation of genes depends on specific interactions of transcription factors (TFs) and regulatory elements on DNA [72,73]. Many TFs were found differentially expressed in our experiments, especially in the co-inoculation of *P. tropica* and *Meloidogyne* spp. (group TBN). In line with the role attributed to *P. tropica* in plant growth, the transcription factor (TF) GRAS was highly upregulated in the TB group. Such a TF has been reported playing roles in plant growth, stress responses and auxin- and gibberellin-mediated signaling pathways [74,75]. Other transcription factors, also differentially expressed in our experiments, like WRKY, MYB and the family ERF, have also been reported associated with defense and growth [76,77,78]. On the other hand, significant and large upregulation of the TF bHLH 094 was observed in groups TN and TBN. A similar scenario has been reported in chickpea plants inoculated with the nematode Pratylenchus [79]. TFs responsive to ethylene were also deregulated, mainly in the TBN group, and have been reported in interactions between tomato and Meloidogyne [80]. Ethylene-mediated defense responses may alter cell susceptibility to nematode infection through the modulation of cellular compound biosynthesis [1].

When differentially expressed genes were analyzed at the gene ontology level, we observed the activation of hydrolase activity on ester bonds when the microbes were inoculated separately or together. These enzymes participate in multiple processes including plant growth, the production of secondary metabolites and hormones, nutrient absorption and responses against biotic and abiotic stress [81,82]. The term ‘hydrolase activity acting on ester bonds’ agglutinates a series of esterase/lipase-encoding genes. Namely, four genes were induced by *P. tropica* alone, eleven by nematodes alone and fourteen in the co-inoculation of the two organisms. Out of those, one esterase/lipase was induced only by *P. tropica*, while another esterase/lipase and a lipase/hydrolase were induced only in the co-inoculation group. Since lipases have been reported to be important in defense responses against pathogens [83,84,85,86,87], it remains possible that the dynamics of lipase expression during *P. tropica*-Meloidogyne co-inoculation favor defense against the nematode. Interestingly, the ontology term ‘fatty acid biosynthetic process’ appeared enriched in the co-inoculation group. Fatty acid biosynthesis requires lipase activity [88] and fatty acids are important in plant defense against pathogens [89]; therefore, the interaction of the bacterium and the nematode seems to enable a feedback loop that promotes defense responses involving fatty acids. In the TN group (nematode alone), ontology terms related to actin and microfilament activity were suppressed. This may occur by active modification of the cell wall mediated by the nematode [90]. In the TBN group, in addition to the hydrolase activity found activated in the TB group, hydrolase activity acting on O-glycosyl compounds was also found among the most activated terms, together with carbohydrate metabolic processes. These are both related to the metabolism of polysaccharides and likely participate in the biosynthesis of cell wall components such as glycan but also in energy mobilization, defense responses, signaling and glycolipid metabolism [86,91]. Another interesting ontology term activated in the double inoculation is related to fatty acid biosynthesis, which has the potential to act as a structural component of membranes and as signal molecules [89].

Finally, ontology terms related to monooxygenase activity and photosynthesis were found suppressed, perhaps suggesting reallocation of resources from photosynthesis towards the production of defense compounds [92,93]. Such a scenario suggests a trade-off between defense and growth in tomato plants [94]. Transcription factors play a key role in reallocating resources from growth to defense in Meloidogyne-infected plants [77,78]. In our case, the plant seems to prioritize defense over growth. This is evident from the suppression of terms related to photosynthesis and other growth-related processes and the upregulation of defense-related processes like cell wall biosynthesis, DNA repair and redox homeostasis [95,96,97]. A very intriguing additional observation is the upregulation of the term ‘carbohydrate metabolic process’, which poses an apparent contradiction with photosynthesis suppression. This suggests a more nuanced response than a simple trade-off between growth and defense. While photosynthesis is suppressed, the activation of glycolysis (Krebs cycle) could exert a compensatory mechanism to provide energy for cellular processes, including defense responses [98]. Moreover, carbohydrate metabolism could be activated to provide precursors for the production of defense compounds, such as callose, lignin and antimicrobial compounds [99,100].

While literature on *P. tropica*’s role in helping tomato plants combat nematode infections is limited or perhaps nonexistent, research on related plant growth-promoting rhizobacteria (PGPR) suggest several mechanisms. PGPR are known to enhance plant defenses against nematodes by triggering systemic resistance, thereby increasing plant resilience [101]. Certain PGPR produce metabolites, such as hydrogen cyanide and siderophores, that exhibit nematicidal properties, as demonstrated for some Pseudomonas species against root-knot nematodes [101]. Additionally, PGPR can outcompete nematodes for nutrients and colonization sites within the rhizosphere, reducing nematode populations and their harmful impact [15]. By promoting nutrient mobilization and overall plant vigor, PGPR can help plants better tolerate and recover from nematode infestations [101]. Future research will illuminate these intriguing possibilities further.

## 4. Materials and Methods

### 4.1. Location

Field experiments were carried out at The Montelindo Farm of the Universidad de Caldas, located in the Santagueda district, municipality of Palestina in Caldas, Colombia. The farm is situated at an altitude of 1010 m.a.s.l. with an average temperature of 22.8 °C, annual precipitation of 2100 mm and average relative humidity of 76%. The preparation of the inoculum of the nematode and of *Paraburkholderia tropica* was conducted, respectively, in the Phytopathology laboratories of the Universidad de Caldas and the Microbiology laboratory of the Research Institute in Microbiology and Agroindustrial Biotechnology of the Universidad Católica de Manizales, located in the municipality of Manizales, in Caldas, Colombia.

### 4.2. Plant Material

Tomato seeds of the Santa Clara variety from Sáenz Fety S.A.S were used; the variety is characterized by its susceptibility to nematodes. Sowing was carried out in 72-locule planting trays on “Sphagnum” peat substrate. Seedlings were fertilized weekly with NPK plus secondary and minor elements, chelated with EDTA. Twenty days after sowing, plants were transplanted into plastic bags with a capacity of 10 kg in soil previously disinfected for nematodes with dazomet (500 g/m) and by solarization for 30 days. The plants were kept on greenhouse benches raised 1.5 m from the ground inside a plastic house under semi-controlled conditions.

### 4.3. Paraburkholderia Tropica Inoculum

The rhizospheric bacteria *P. tropica* (1 × 10^8^ CFU/mL), native to the department of Caldas, was provided by the Microorganism Collection of the Universidad Católica de Manizales (accession GIBI_0183). The inoculum was preserved in 30% glycerol at −80 °C. For reactivation, bacteria were thawed and plated on LB agar and incubated at 28 °C for 24 h. For inoculum amplification, three bacterial colonies were inoculated into a Schott flask containing 30 mL of LB nutrient broth in an orbital shaker incubator set to 112 rpm and 30 °C. Bacterial growth was allowed until the culture reached an optical density between 0.9 and 1.0 at 600 nm, equivalent to a concentration of 1 × 10^8^ CFU/mL. The procedure was carried out in the Microbiology laboratory of the Agroindustrial Microbiology and Biotechnology Research Institute of the Universidad Católica de Manizales.

### 4.4. Inoculum of Meloidogyne spp.

Because pure cultures of *Meloidogyne incognita* are poor colonizers of the tomato radical area, a field isolate enriched in *M. incognita* was used instead. Samples were taken from the roots of tomato plants infected by nematodes. The method proposed by Hussey and Baker was used to extract eggs [102], which were incubated to obtain infective juveniles (J2). The suspension containing J2 nematodes was diluted in water to achieve an approximate concentration of 1000 juveniles per 50 mL which were inoculated around the seedling 15 days after transplanting.

### 4.5. Evaluated Treatments

Three plants were used for each treatment. Treatment one corresponds to the control plants without application of microbes (TC). In treatments two (TB) and four (TBN), two foliar applications of *P. tropica* were conducted at a concentration of 1 × 10^8^ CFU: the first one on the day of transplant and the second one fourteen days after transplanting. For treatments three (TN) and four (TBN), nematodes were inoculated in the rhizosphere area of the plant fifteen days after sowing (DAS). For treatment four (TBN), this inoculation took place 24 h after the second application of *P. tropica*. All plants were fertilized as indicated above and plant height and number of leaves were recorded weekly from transplant until the first sampling.

The sampling of systemic leaves (three leaves per plant) for transcriptome analysis was conducted 48 h after inoculation with the nematode in all groups. Samples were collected after 5:30 p.m., snap-frozen in liquid nitrogen and then stored at −80 °C. The samples were lyophilized in the laboratory of the Institute of Biotechnology and Agroindustry of the Universidad Nacional de Colombia and sent to Novogene (Hong Kong) for RNAseq library construction and sequencing.

### 4.6. Library Construction and Sequencing

RNAseq libraries were constructed using the Illumina TruSeq RNA Library Prep Kit v2 (Illumina, San Diego, CA, USA) according to the manufacturer’s instructions. Briefly, total RNA was extracted with TRIzol reagent and precipitated in ethanol. Polyadenylated mRNA transcripts were captured using dT-oligos conjugated to magnetic beads. Enriched mRNA was end-repaired, A-tailed and ligated to linkers that provided the annealing site for sequencing oligos harboring barcodes that distinctly labeled each sample. Libraries were pooled at equimolar concentrations and sequenced at 10 pM in a HiSeq 2500 instrument (Illumina, San Diego, CA, USA) with a paired-end protocol of 150 cycles that included on-instrument demultiplexing. Samples were sequenced at an average depth of 15.44 million reads.

### 4.7. Quality Control

Sequences were end-trimmed with the ea-utils suit using the fastq-mcf (https://expressionanalysis.github.io/ea-utils/, 1 March 2024) program with a quality score threshold of 20 and allowing a minimal length of 100 bp. This analysis resulted in only a small reduction in the number of reads, bringing the average of trimmed reads to 14.47 million per sample.

### 4.8. Transcriptome Assembly

Sequences from all libraries were concatenated for end1 and end2 and subjected to assembly with rnaSPAdes [48] using a paired-end algorithm. Assembled contigs were used to predict transcripts with the algorithms TransDecoder.LongestOrfs and TransDecoder.Predict (https://github.com/TransDecoder/TransDecoder, 23 April 2024). These algorithms, respectively, screen for the longest ORF contained in a contig and then screen for motifs that are typical in protein sequences and contained in the Pfam database. Finally, a log-likelihood score is used to rank the hexamers in the predicted ORF. Only complete predicted transcripts (including a start codon and a stop codon) were selected for annotation, while 5′ and 3′ moieties were discarded. Such complete predicted transcripts were annotated with Trinotate [51], which uses a large body of databases including Pfam, KEGG, EggNOG, Swissprot, Uniprot and Gene ontology.

### 4.9. Transcript Quantification and Differential Expression Analysis

Transcripts were quantified using the Kallisto program (v0.50.0) with bias correction [103]. Results were parsed with the script ‘extract_kallisto_results.sh’ which yields files all_samples_counts.tsv and all_samples_tpms.tsv, containing raw and normalized counts (reads per million per transcript kilobase). Raw counts were used for differential expression analysis with the R package DESeq2 [104]. DESeq2 calculates a size factor proportional to the fraction of each library that could be mapped using Kallisto to account for differences in library size. Gene dispersion was assessed as a proxy of variability in expression counts across replicates transcending a Poisson distribution. For differential expression, a negative binomial distribution model was used which incorporates the normalized counts and dispersion estimates to test for differential expression. The model was fitted to the data and hypothesis testing was performed to compare conditions. A transcript was considered differentially expressed if the adjusted *p* Value < 0.05 and the absolute value of |log2FC| > 1.

### 4.10. Gene Ontology Analysis

Gene ontology analysis was conducted using the R package clusterProfiler [105], but using a custom script that included the ontology classification for the canonical tomato genes and the novel genes which were classified with Trinotate [51]. Over-representation analysis was conducted using the general algorithm ‘enricher’; pValues were corrected by applying the Benhamini–Hochberg test to statistically significant enriched terms having a q Value < 0.1.

## 5. Conclusions

The results presented here suggest that *Paraburkholderia tropica* effectively primes defense responses against *Meloidogyne* spp. in tomato plants. These responses are multilayered, encompassing various mechanisms that enhance the plant’s defense robustness. In addition to the transcripts included in the tomato reference transcriptome used here, we managed to assemble a large number of putative novel transcripts that provide additional insights into the mechanism underlying plant–nematode molecular interactions and may serve as candidates for future studies. Practically, our findings support incorporating *P. tropica* into integrated management strategies for nematode-induced diseases and also lay the groundwork for further research initiatives related to plant–pathogen interactions and breeding programs to confer resistance against nematodes. Finally, we would like to state that, to the best of our knowledge, this is the first report on the interaction of *P. tropica* and *Meloidogyne* spp. in tomato and therefore it opens a myriad of research avenues on the biology of this pathosystem.

## Figures and Tables

**Table 1:** Summary of differentially expressed transcripts and gene ontology terms found over- or under-represented.

	TB	TN	TBN
Number differentially expressed (DE) transcripts	124	203	560
Number upregulated transcripts	100	173	414
Number downregulated transcripts	24	30	146
Number upregulated resistance genes	0	3	6
Number DE transcription factors [up/down]	5/1	4/3	15/5
Number DE protein kinases [up/down]	4/2	5/1	30/9
Over-represented GO terms	GO:0004867 GO:0016788	GO:0016788 GO:0016614	GO:0016788 GO:0016614 GO:0008725 GO:0004553 GO:0005975 GO:0016747 GO:0045454 GO:0006633 GO:0006284 GO:0016760 GO:0030244
Under-represented GO terms	GO:0048573 GO:0045814 GO:0004553 GO:0000122 GO:0006415	GO:0003779 GO:0000146 GO:0003723 GO:0030048 GO:0007131 GO:0045944 GO:0008654 GO:0000122 GO:0000785 GO:0016049 GO:0000981 GO:0003774 GO:0007015 GO:0043565 GO:0010215 GO:0016459	GO:0019684 GO:0009772 GO:0045156 GO:0016705

## Data Availability

Raw sequencing data have been deposited to the Short Reads Archive (SRA) database of NCBI and are freely available under accession number PRJNA1057628. All bioinformatics procedures and scripts used are publicly available from our github repository (https://github.com/paraburkh/RNAseq_paraburkh).

## Supplementary Materials

The following supporting information can be downloaded at: https://www.mdpi.com/article/10.3390/ijms252312584/s1.
