# Supplementary material for: Paraburkholderia tropica Primes a Multilayered Transcriptional Defense Response to the Nematode Meloidogyne spp. in Tomato
Source: Int J Mol Sci. 2024 Nov 23;25(23):12584. doi: 10.3390/ijms252312584 (PMC11641443; doi:10.3390/ijms252312584)
Supplement: Supplementary file 1 [file ijms-25-12584-s001.zip › ijms-3313282-supplementary figures.pdf]

International Journal of *Molecular Sciences*

MDPI

Article

Paraburkholderia tropica primes a multilayered transcriptional defense response to the nematode *Meloidogyne* spp. in tomato

Carolina González-Cardona [1], Walter Ricardo López [2], Juan Jovel [1,3], Mauricio Soto-Suárez [1,4] and Nelson Ceballos-Aguirre [1*]

[1] Universidad de Caldas. Doctorado en Ciencias Agrarias, Facultad de Ciencias Agropecuarias. Calle 65 No. 26-10, 170003. Manizales, Caldas, Colombia carolina.gonzalez@ucaldas.edu.co, nelson.ceballos@ucaldas.edu.co

[2.] Universidad Nacional de Colombia sede Manizales. Departamento de Física y Química. Facultad de Ciencias Naturales. Km 9 vía aeropuerto La Nubia 170003, Manizales, Caldas, Colombia. wrlopez@unal.edu.co

[3] University of Calgary. Faculty of Veterinary Medicine. Cal Wenzel Precision Health Building, CWPH 1E08, Calgary, AB T2N 4Z6. Canadá. juan.jovel@ucalgary.ca

[4] Corporación Colombiana de Investigación Agropecuaria-AGROSAVIA. Km 14 Vía, Mosquera, Bogotá, 250047, Mosquera, Cundinamarca, Colombia. msoto@agrosavia.co

* Correspondence: author: nelson.ceballos@ucaldas.edu.co

Supplementary Materials

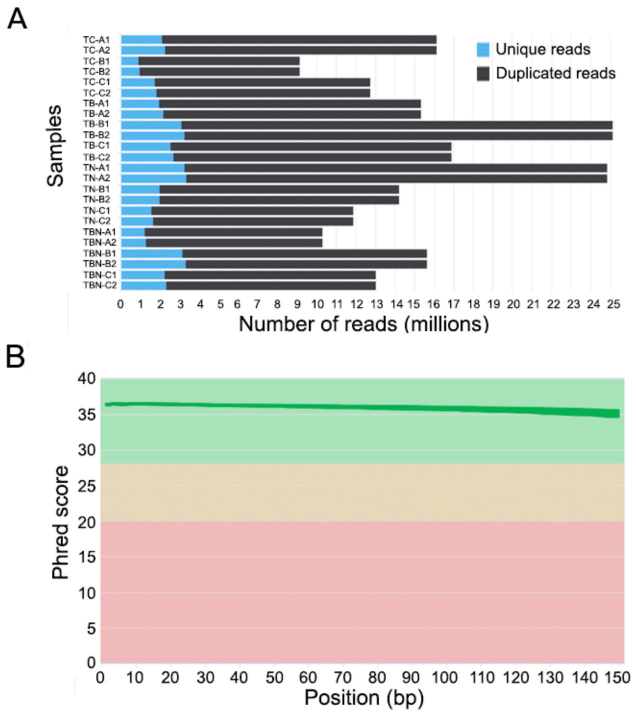

Figure S1. Size and quality of NGS libraries. (A) Number of total reads and unique library and end. (B) Average quality of reads before trimming.

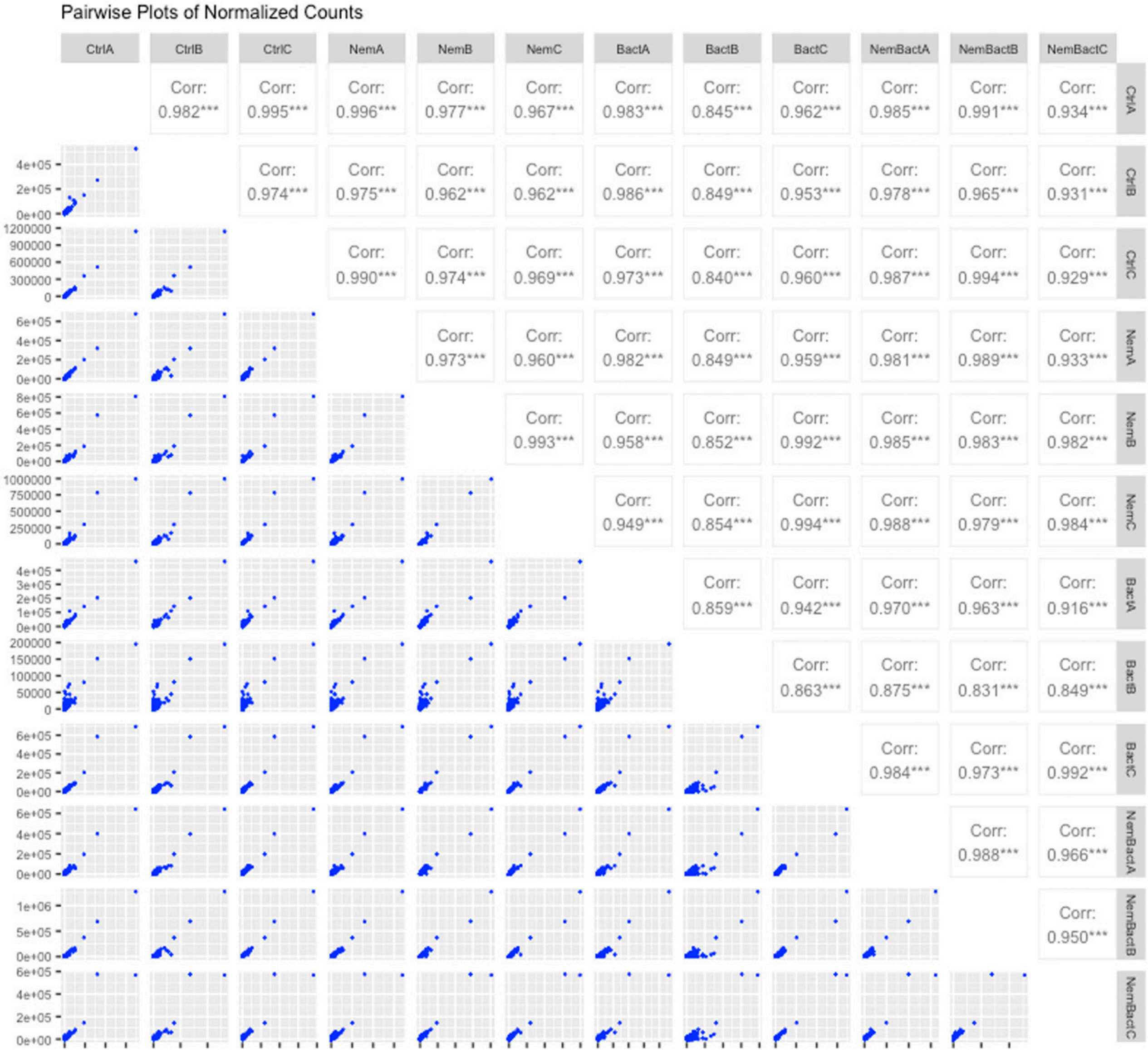

Figure S2. Correlation coefficients of gene counts for all libraries sequenced. Counts for all genes were plotted in a pairwise manner (each sample against all other samples). Correlation coefficients greater than 0.8 and 0.9 are considered good and excellent quality libraries, respectively.

Resistance gene	TB	TN	TBN
Disease resistance protein I2	0	0	7.942502
Putative late blight resistance protein homolog R1B-12	0	0	7.742254
Tobacco mosaic virus resistance-2	0	4.819063	5.31215
Late blight resistance protein	0	0	2.877917
Pleiotropic drug resistance protein 4	0	1.530274	2.256487
Disease resistance protein (TIR-NBS-LRR class) family	0	0	1.725911
Disease resistance protein	0	7.176082	0

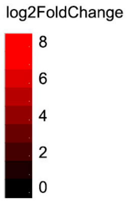

Figure S3. Heatmap with name and fold change of DE transcripts derived from resistance genes.

Transcription factor	TB	TN	TBN
bHLH transcription factor 094	0	8.081366457	9.913969929
Transcription factor PERIANTHI	8.003124952	0	8.792080542
Transcription factor TCP13	0	0	7.423725446
Ethylene-responsive transcription factor TIN	0	0	7.211489839
Transcription factor PAR2	0	0	7.054882339
Ethylene-responsive transcription factor SHINE 2	0	0	4.618605055
Basic-leucine zipper (BZIP) transcription factor family protein	0	0	3.244580166
transcription factor MYC	1.852230545	1.634454289	2.098385537
TCP transcription factor 14	0	0	1.815463424
T-box transcription factor	0	0	1.745825762
bHLH transcription factor 012	0	0	1.720516934
Transcription factor UNE10	0	0	1.541486274
Transcription factor MYC/MYB domain-containing protein	0	0	1.236743014
Ethylene-responsive transcription factor RAP2.1	0	1.627654691	0
GATA transcription factor (K4BQ39_SOLLC)	0	-2.441479576	0
GATA transcription factor (K4C2T6_SOLLC)	1.765307435	0	0
GATA transcription factor 23	0	-1.59383372	0
R2R3MYB transcription factor 3	0	1.529753056	0
Transcription factor GRAS	8.270471841	0	0
myelin transcription factor	8.280279071	0	0
WRKY transcription factor 34	0	0	-1.827322458
Ethylene-responsive transcription factor 5	0	0	-2.001496791
Transcription factor atf1	0	0	-2.110229689
Myb-related transcription factor LBM2	0	0	-2.978532315
Ethylene-responsive transcription factor (A0A2G3BP26_CAPCH)	0	0	-8.298089765

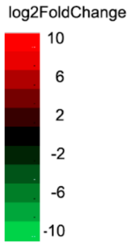

Figure S4. Heatmap with name and fold change of DE transcripts encoding transcription factors.

Protein kinase	TB	TN	TBN
Serine/threonine-protein kinase SRK2I	0	0	10.25379
Receptor-like protein kinase THESEUS.1	0	0	9.95401
Receptor-like protein kinase (A0A2R6QLC9_ACTCH)	0	0	8.020485
Leucine-rich receptor-like protein kinase family protein	0	7.674105	7.718933
Protein kinase domain (A0A200QIT5_9MAGN)	0	0	7.572666
Cyclin-dependent protein kinase inhibitor SMR8	0	0	6.538902
Leucine-rich repeat receptor protein kinase (A0A2R6RQU4_ACTCH)	0	0	6.314742
Serine/threonine-protein kinase Nek5	0	0	6.253746
Leucine-rich repeat protein kinase family protein (Q9FMI2_ARATH)	0	0	5.295095
LRR receptor-like kinase family protein (A0A072VP04_MEDTR)	0	0	4.380764
Phosphatidylinositol 4-phosphate 5-kinase (K4B029_SOLLC)	0	3.675788	3.513369
"Receptor protein kinase, putative (B9T1Q4_RICCO)"	0	0	3.354075
Protein kinase domain (A0A200QFW1_9MAGN)	3.408828	3.291159	3.335126
"Receptor protein kinase, putative (A0A061FG24_THECC)"	0	0	3.035834
G-type lectin S-receptor-like serine/threonine-protein kinase LECRK3	0	0	2.470771
receptor-like kinase TMK3	0	0	2.435191
Calmodulin-binding receptor-like cytoplasmic kinase 2	0	0	2.171533
Serine/threonine-protein kinase D6PKL2	0	0	2.104032
Leucine-rich receptor-like protein kinase family protein (A0A0K9NNG1_ZOSMR)	0	0	1.894579
Receptor-like protein kinase (A0A2R6QLC9_ACTCH)	0	0	1.875982
Serine/Threonine-kinase pakA-like protein (AT3G15095.1)	1.656372	0	1.823405
MOB kinase activator family	0	0	1.570368
Receptor-like kinase (G7K0V8_MEDTR)	0	0	1.561866
Receptor-like protein kinase THESEUS.1	0	0	1.533689
Ovary receptor kinase 27	0	0	1.533571
Protein kinase domain (A0A200Q940_9MAGN)	0	0	1.525492
Protein kinase 2B	0	0	1.493073
Protein kinase domain (A0A200QBC6_9MAGN)	0	0	1.40058
Protein kinase domain (A0A200QZD6_9MAGN)	0	0	1.184219
Protein kinase G11A	0	0	1.009392
G-type lectin S-receptor-like serine/threonine-protein kinase (A0A2G2WZR9_CA	8.788198	0	0
Leucine-rich repeat receptor-like tyrosine-protein kinase (A0A2R6R578_ACTCH)	7.297535	0	0
Protein kinase superfamily protein (AHRD V3.3 *-* O80962_ARATH)	-8.374313	0	0
Cysteine-rich receptor-like protein kinase 10 (A0A2G2XFS8_CAPBA)	0	7.612463	0
Serine/threonine-protein kinase-like protein ACR4 (A0A2G2YFK6_CAPAN)	0	4.955935	0
"GTP pyrophosphokinase, (P)ppGpp synthetase II Guanosine-3',5'-...	0	0	-1.195443
5'-AMP-activated protein kinase subunit beta-2 (A0A1J3HGR2_NOCCA)	0	0	-1.278737
Wall-associated receptor kinase-like 20	0	0	-1.458787
Xylulose kinase (A0A2G2X084_CAPBA)	0	0	-1.868347
Protein kinase family protein (A0A1P8BDJ0_ARATH)	0	0	-1.963966
G-type lectin S-receptor-like serine/threonine-protein kinase (A0A1U8GJN2_CA	0	0	-2.000649
Receptor-like protein kinase (AHRD V3.3 *** A0A2K3NJB3_TRIPR)	-2.694089	0	-3.564096
auxin-regulated dual specificity cytosolic kinase	0	0	-8.076268
Protein kinase superfamily protein (A0A2U1KAY9_ARTAN)	-10.18635	0	-10.40239

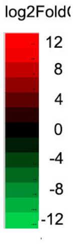

Figure S5. Heatmap with name and fold change of DE transcripts encoding protein kinases.

Novel transcripts	TB	TN	TBN
Serine/threonine-protein kinase SRK2I	0	0	10.25378775
MLO-like protein 6	0	0	8.737482927
Probable sulfate transporter 3.3	0	0	8.676805393
Dehydrin COR47	0	0	7.851533097
Probable galacturonosyltransferase-like 2	-7.772558407	0	7.808915669
Putative late blight resistance protein	0	0	7.7422543
Probable glycosyltransferase STELLO1	7.496811557	0	7.67987323
Auxin-responsive protein SAUR66	7.901607599	7.315946866	7.596253943
Phytosulfokine receptor 1	0	7.41455235	7.534294852
Superoxide dismutase [Cu-Zn] 2	0	7.132495976	7.524558035
Transcription factor TCP13	0	0	7.423725446
SPX domain-containing protein 1	0	0	7.393303706
Cytosolic purine 5'-nucleotidase	0	0	6.97849869
Cyclin-dependent protein kinase inhibitor SMR8	0	0	6.538902328
GDSL esterase/lipase At5g45670	0	5.567944823	6.341622387
Serine/threonine-protein kinase Nek5	0	0	6.253745514
Glycine-rich protein	7.081169248	5.572905993	5.698893394
Ethylene-responsive transcription factor SHINE 2	0	0	4.618605055
"Glutathione reductase, chloroplastic"	0	0	4.399207241
DNA ligase 4	0	0	4.20426167
"Acyl-coenzyme A thioesterase 4, mitochondrial"	0	0	3.840360923
Putative glycosyltransferase 7	0	0	3.629921396
Probable pectate lyase 15	2.679690271	2.909638313	3.278231521
Protein HOTHEAD	0	2.08154007	2.725807855
Nucleolar GTP-binding protein 1	0	0	2.453366444
Agamous-like MADS-box protein AGL19	0	2.358511106	2.440033916
PLAT domain-containing protein 2	2.585443846	0	2.307177406
Ras-related protein RABA1f	2.455354609	2.272752527	2.291584508
Fimbrin-2	0	0	2.191729685
F-box protein At5g46170	0	0	2.12779514
Serine/threonine-protein kinase D6PKL2	0	0	2.104031683
Small ribosomal subunit protein uS14m	0	0	1.909182911
Tubulin alpha chain	0	0	1.81347047
Subtilisin-like protease 4	0	0	1.800582449
Protein SULFUR DEFICIENCY-INDUCED 2	0	1.388109267	1.680270199
"Alanine aminotransferase 2, mitochondrial"	0	0	1.631637851
"Superoxide dismutase [Fe], chloroplastic"	1.492312115	1.182387543	1.613030589
ABC transporter B family member 2	0	0	1.575045858
Transcription factor UNE10	0	0	1.541486274
Large ribosomal subunit protein uL29c	0	0	1.384234316
Protein DEHYDRATION-INDUCED 19	0	0	1.22182852
Basic leucine zipper 61	0	0	1.069349204
"Peptide chain release factor PrfB1, chloroplastic"	-1.523075896	0	-1.215069007
Thioredoxin H2	0	0	-1.298102403
QWRF motif-containing protein 2	0	0	-1.298921619
Zinc finger protein CONSTANS-LIKE 5	0	0	-1.360303725
Wall-associated receptor kinase-like 20	0	0	-1.458787014
Cytochrome P450 94A1	0	0	-1.615541544
Myosin-9	0	0	-1.826629533
Cyclic nucleotide-gated ion channel 4	0	0	-1.872887185
F-box protein CPR1	0	0	-2.059017345
Protein ALP1-like	-4.10462199	0	-2.579711789
Glycine-rich RNA-binding protein 3	0	0	-3.290142998
Cytochrome P450 89A2	0	0	-7.409914348
Carbon catabolite repressor protein 4 homolog 6	0	0	-7.47640756
Protein NETWORKED 4A	0	-7.456048073	-7.672561165
Probable protein phosphatase 2C 60	0	0	-7.955035173
Putative RNA polymerase II subunit B	0	0	-7.994845752
Beta-glucosidase 18	0	0	-8.472306325
"Pyruvate dehydrogenase E1 component subunit alph"	-8.145418675	-8.270958742	-8.486343
RNA-binding protein 2	0	0	-9.525364249
Probable uridine nucleosidase 1	0	0	-21.73608147
"NADP-dependent malic enzyme, chloroplastic"	0	-8.22265077	0
Abscisic stress-ripening protein 2	0	1.476503629	0
ARF guanine-nucleotide exchange factor GNOM	0	7.746697075	0
Auxin response factor 18	8.108464998	8.243373831	0
Maturase K	-1.601934772	0	0
NAC domain containing protein 52	-3.793612872	0	0
PLAT domain-containing protein 1	0	1.62420429	0
Probable metal-nicotianamine transporter YSL7	8.229186354	0	0
Protein EIN4	0	7.101413522	0
Protein TIC 214	-1.903082543	0	0
Splicing factor 3B subunit 1	0	7.030031802	0
Transcription factor atf1	0	-2.110229689	0
Tropinone reductase homolog At5g06060	0	1.512520595	0

log2FoldChange: 12, 8, 4, 0, -8, -16, -24

Figure S6. Heatmap with name and fold change of DE putative novel transcripts.

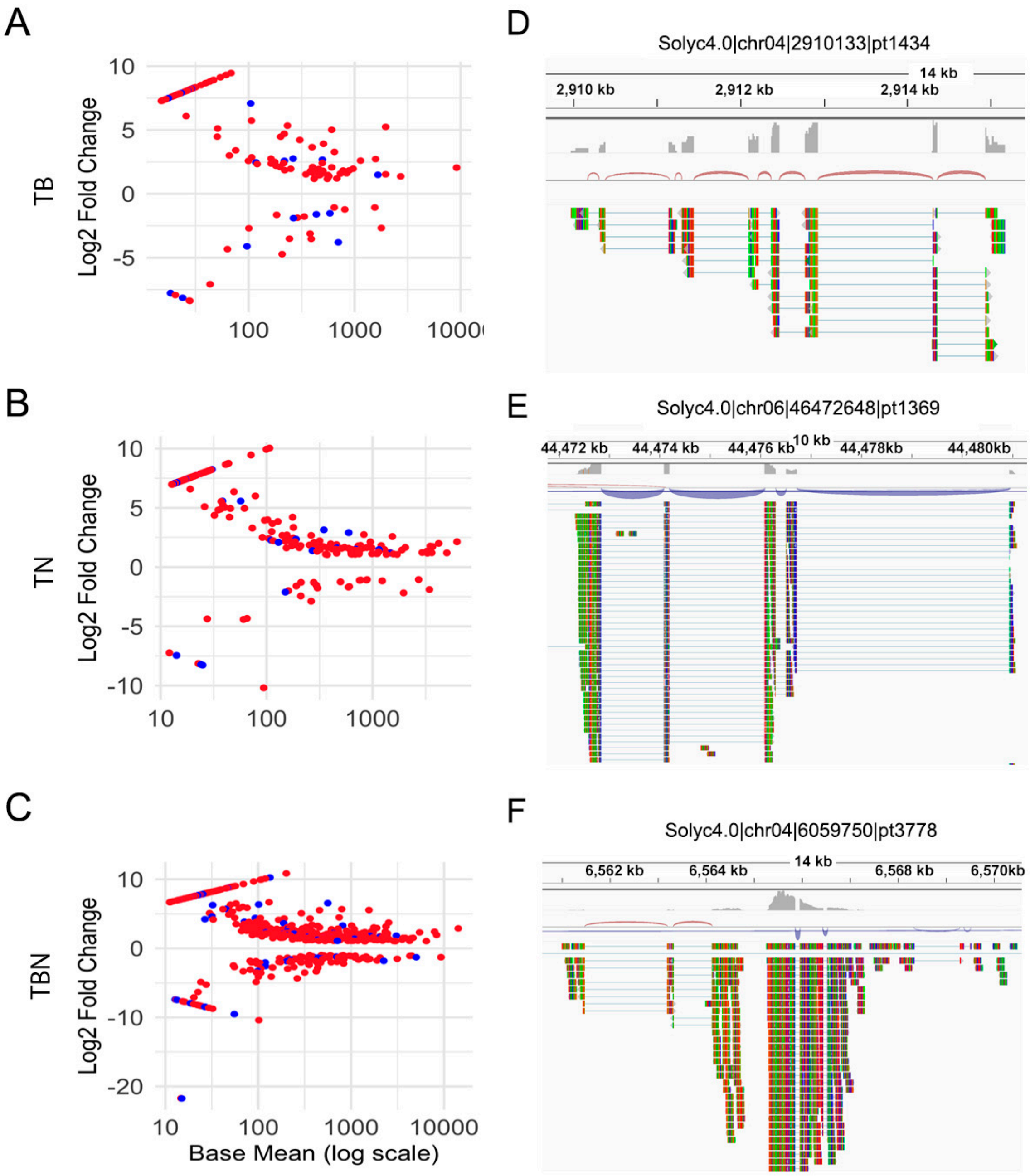

Figure S7. Supporting evidence for putative novel transcripts. MA plot depicting base mean and log2fold change for TB (A), TN (B) and TBN (C). Red dots represent transcripts in the reference transcriptome, blue dots represent putative novel transcripts. Examples of spliced alignment of putative novel transcripts for TB (D), TN (E) and TBN (F).

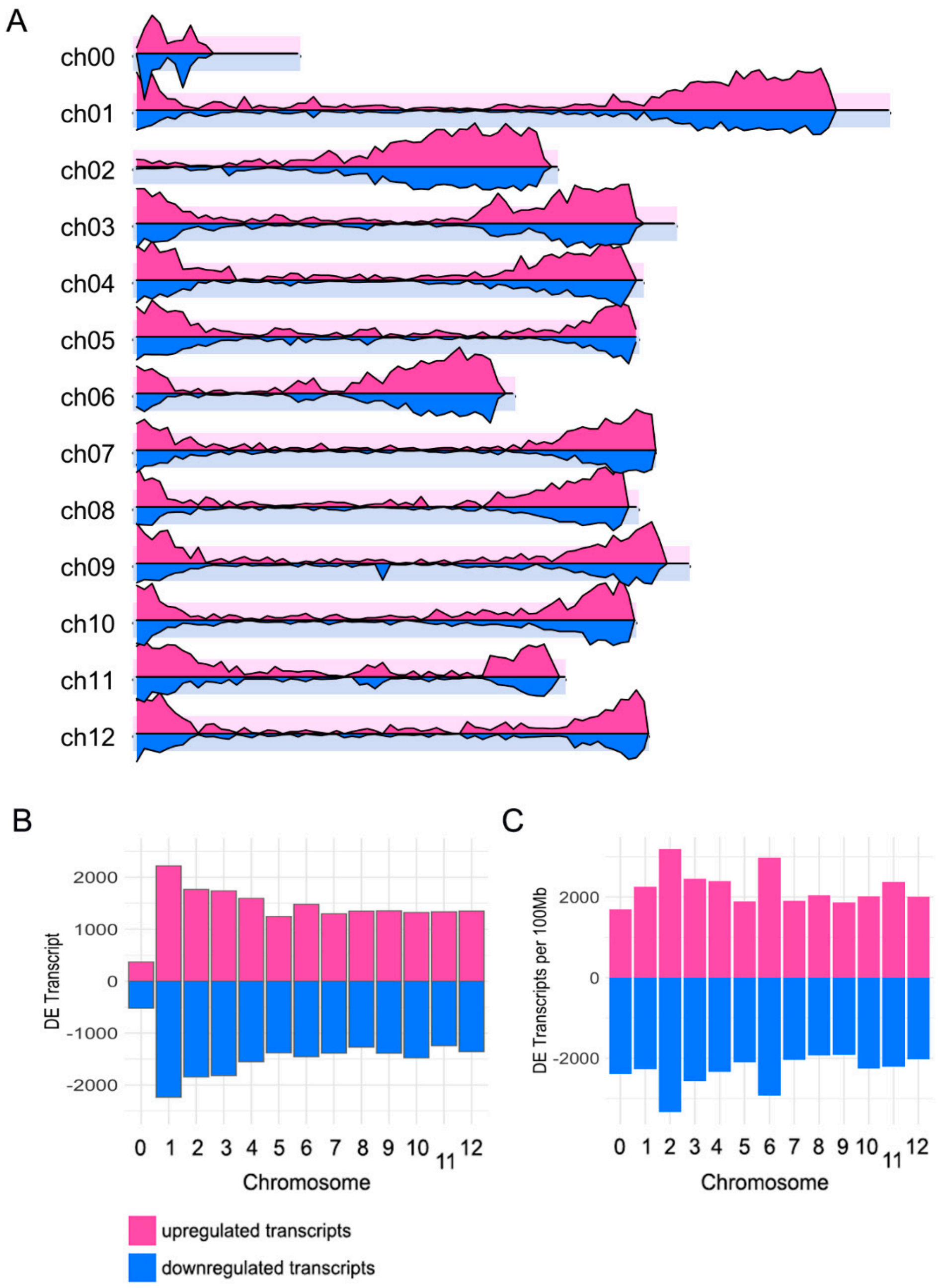

Figure S8. Distribution of transcripts along the tomato genome. (A) Density plot of all transcripts of tomato along the genome. (B) Counts of transcripts per chromosome. (C) Normalized (per 100 mB) number of transcripts per chromosome.

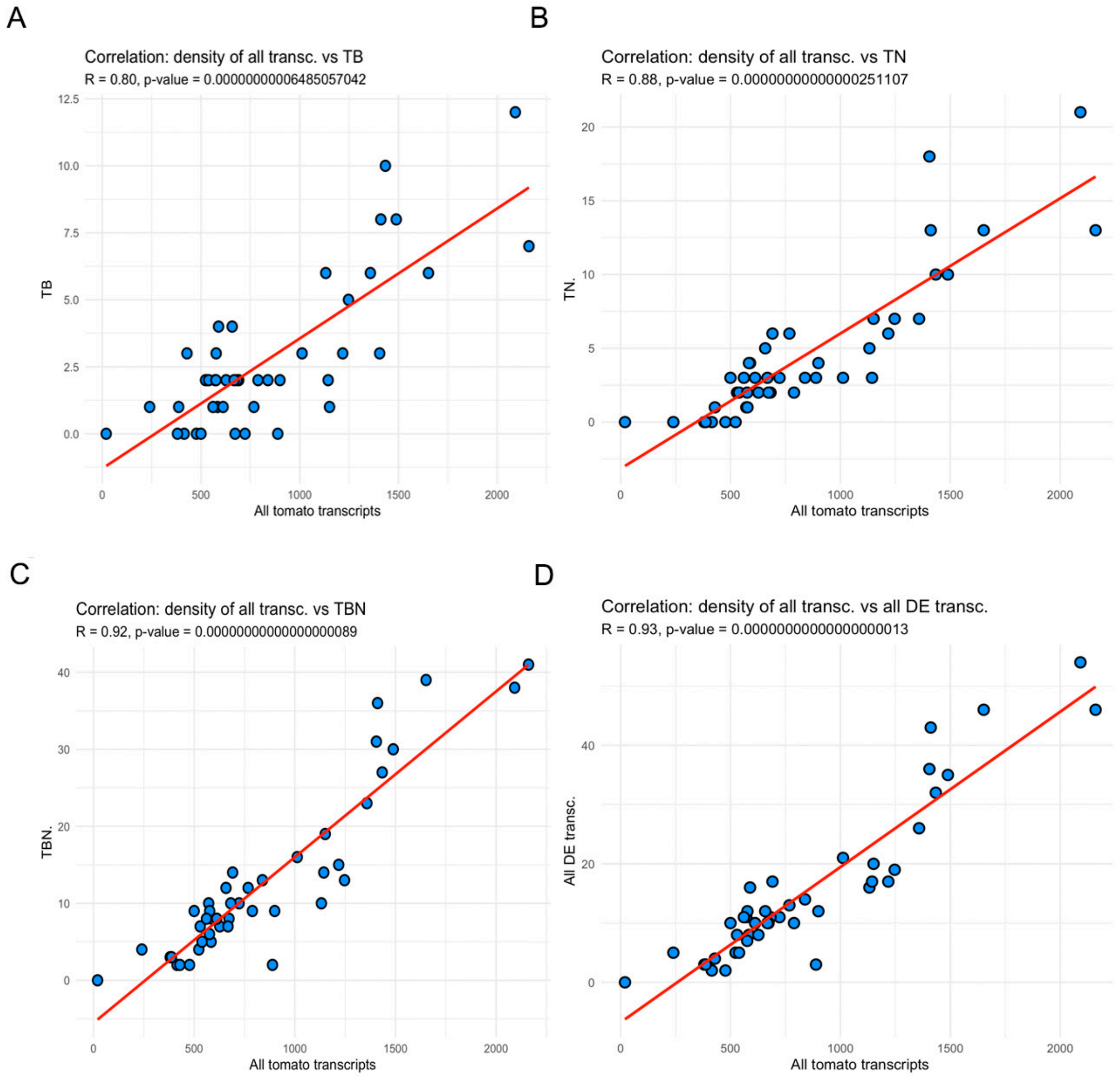

Figure S9. Pearson correlation analysis between the general distribution of genes along the tomato genome and DE transcripts in each of our treatments. All chromosomes were concatenated and each chromosome was divided in four segments. The overall density of genes in the tomato genome or those found DE in each treatment were correlated. r coefficient and p-value are indicated in each case for TB (A), TN (B), TBN (C) or all of them together (D).
